# Reduced brain activity in female patients with non-alcoholic fatty liver disease as measured by near-infrared spectroscopy

**DOI:** 10.1371/journal.pone.0174169

**Published:** 2017-04-04

**Authors:** Atsushi Takahashi, Soichi Kono, Akira Wada, Sachie Oshima, Kazumichi Abe, Hiromichi Imaizumi, Masashi Fujita, Manabu Hayashi, Ken Okai, Itaru Miura, Hirooki Yabe, Hiromasa Ohira

**Affiliations:** 1 Departments of Gastroenterology, Fukushima Medical University School of Medicine, Fukushima, Japan; 2 Neuropsychiatry, Fukushima Medical University School of Medicine, Fukushima, Japan; 3 Department of Neuropsychiatry, The University of Tokyo Hospital, Tokyo, Japan; Chiba Daigaku, JAPAN

## Abstract

Patients with non-alcoholic fatty liver disease (NAFLD) have impaired health-related quality of life including physical and mental state. Near-infrared spectroscopy (NIRS) is a useful tool for evaluation of brain activity and depressive state. This study aimed to determine the brain activity of female NAFLD patients using NIRS. Cerebral oxygenated hemoglobin (oxy-Hb) concentration during a verbal fluency task (VFT) was measured using NIRS in 24 female NAFLD patients and 15 female healthy controls. The Center for Epidemiologic Studies Depression Scale (CES-D) questionnaire was administered to both groups before NIRS. There was no significant difference in CES-D score between groups. However, the oxy-Hb concentration and number of words during the VFT were less in NAFLD compared to healthy controls. The mean value of oxy-Hb concentration during 0–60 s VFT in the frontal lobe was also smaller in NAFLD patients compared to healthy controls (0.082 ± 0.126 vs. 0.183 ± 0.145, *P* < 0.001). Cerebral oxygen concentration is poorly reactive in response to VFT in female NAFLD patients. This may indicate an association between decreased brain activity and NAFLD regardless of depression.

## Introduction

Although some patients with non-alcoholic fatty liver disease (NAFLD) have depression in addition to a positive association between depression and liver histological severity of NAFLD [1–4], other studies have found no association between depression and NAFLD [5–6]. This discrepancy may result from differences in evaluation methods for depression. On the other hand, patients with NAFLD have impaired health-related quality of life. The scores of physical and mental domains in the 36-item Short Form Health Survey (SF-36) questionnaire and Chronic Liver Disease Questionnaire have been observed to be low in patients with NAFLD, including patients with non-alcoholic steatohepatitis (NASH), compared to the general population or to patients with hepatitis B [7–9]. These results imply that NAFLD patients have depressive state even without apparent depression.

The Center for Epidemiologic Studies Depression Scale (CES-D) and the Patient Health Questionnaire (PHQ-9) are commonly used self-report depression scales [10–11]. These methods are easy and useful tools for screening of depression, but they lack objectivity because they are self-report scales. Neurobiological imaging studies of verbal fluency, such as positron emission tomography (PET) and functional magnetic resonance imaging (fMRI), have been proposed to overcome the limitations of depression diagnosis [12].

Near-infrared spectroscopy (NIRS) can measure cerebral blood volume as an oxygenated hemoglobin (oxy-Hb) concentration. The front-temporal NIRS signal has been proposed as a supportive tool in assisting the diagnosis of major psychiatric disorders with depressive symptoms in addition to evaluation for brain activity [13–15]. NISR is also convenient in that it is easily performed and is low-cost in comparison with PET or fMRI.

We hypothesized that evaluation for brain activity using NIRS can elucidate the difference between NAFLD patients and healthy individuals. In the present study, we investigated possible latent abnormality of brain function using NIRS in female NAFLD patients. Female patients were evaluated because female sex is an independent predictor of depression in NAFLD [2].

## Materials and methods

### Participants

In present study, we recruited a twenty-four Japanese female NAFLD patients without psychiatric disorders including depression from Fukushima University Hospital, and 15 age-adjusted Japanese healthy females between December 2013 and July 2016. The diagnosis of NAFLD was based on the Asia-Pacific Working Party guidelines for the assessment and management of NAFLD [16]. Fatty liver was detected by ultrasonography in the absence of other causes of chronic liver disease (e.g., hepatitis C antibody negative, hepatitis B surface antigen negative, and alcohol consumption < 20 g/day). No subjects took any sleeping pills, antidepressants or antipsychotics. Diabetes was defined as fasting plasma glucose ≥ 126 mg/dL, hemoglobin A1c (HbA1c) ≥ 6.5%, or the self-reported use of antihyperglycemic agents. Liver cirrhosis was diagnosed based on the clinical features, laboratory and imaging findings. Dyslipidemia was defined as fasting low-density lipoprotein cholesterol (LDL-C) ≥ 140 mg/dL, fasting triglycerides (TG) ≥ 150 mg/dL, fasting high-density lipoprotein cholesterol (HDL-C) < 40 mg/dL, or the self-reported use of antidyslipidemic agents. Hypertension was defined as systolic blood pressure (BP) > 140 mmHg, diastolic BP > 90 mmHg, or the self-reported use of antihypertensive agents. This study was approved by the Ethics Committee of Fukushima Medical University (#1735). All subjects provided written informed consent to participate in the study.

### Activation task

A verbal fluency task (VFT) was used to estimate oxy-Hb activation in the frontal to temporal lobes. Briefly, participants were instructed to say as many Japanese words starting with a designated syllable as possible for 20 s. The task was repeated 3 times during the 60-s task periods.

### NIRS measurement

**Fig 1:**
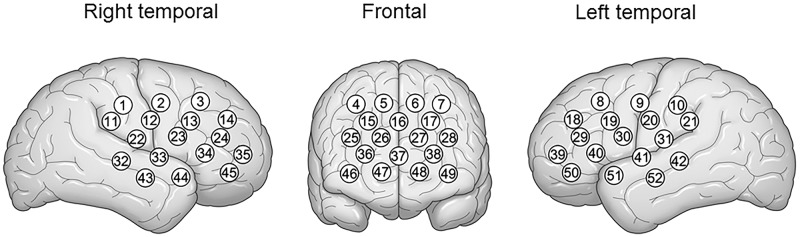
Location of the 52-channel Near-Infrared Spectroscopy (NIRS) probes in the right temporal, frontal, and left temporal brain regions.

NIRS measurement was performed as described previously [14]. Concentrations of oxy-Hb and deoxy-Hb were measured using a 52-channel NIRS system (ETG-4000; Hitachi Medical Co., Tokyo, Japan) using 2 wavelengths (695 and 830 nm) of infrared light (Fig 1) during a 10-s pre-task period, a 60-s activation period and a 55-s post-task baseline period. Reflected light was measured by pairs of detector probes positioned 3.0 cm away and each measuring area was defined as a channel. Each lobe of the brain was covered by the 52 channels. The rate of data sampling was 0.1 seconds and obtained data were analyzed using integral mode; the mean during a 10-s pre-task period was determined as the pre-task baseline, and the mean over the last 5-s of the post-task period was determined as the post baseline, and linear fitting used to the data between these two lines. We applied the algorithm developed by Takizawa et al [14] to automatically reject the data with artifacts. Data were expressed as forms of wave and topographic images. The mean of the increases in oxy-Hb concentrations in seven channels diagnostic for mental disorders [13–14] (channels 36–38 and 46–49) was calculated and compared between the NAFLD patients and the controls (S1 File).

### Questionnaires

The CES-D questionnaire was administered to assess depressive symptoms before NIRS measurements. The CES-D is one of the most common screening questionnaires, consisting of 20 items about feelings (e.g., happy, fearful, depressed) and behaviors (e.g., crying, eating, sleeping) [10]. The total score ranges from 0 to 60; a score of 16 or higher indicates depressed mood. Sleep quality was evaluated by the Pittsburgh Sleep Quality Index (PSQI) before NIRS. The PSQI is one of the most widely used standardized questionnaires for evaluating sleep quality. Nineteen questionnaire items generate seven component scores: sleep quality, sleep latency, sleep duration, habitual sleep efficiency, sleep disturbances, use of sleeping medication, and daytime dysfunction [17].

### Statistical analysis

Continuous data are expressed as median (interquartile range). The two groups (NAFLD patients and healthy controls) were compared using the Mann-Whitney U test. Sample size and power were calculated based on previous our study [18], power = 0.8, α = 0.05. If there were missing values, statistical analysis was performed using available data. Correlations between laboratory variables were measured using Spearman’s correlation coefficient. Statistical analyses were performed using SPSS 17.0 for Windows (SPSS, Inc., Chicago, IL, USA). MATLAB R2011 (MathWorks Inc., Natick, MA) software and Prism 6.0 software (GraphPad Software, Inc). A *P* value < 0.05 was considered significant.

## Results

The clinical characteristics of the NAFLD patients and healthy controls are shown in Table 1 (S1 File). There were no differences in age, CES-D score, or PSQI score, respectively. Body mass index (BMI) was significantly higher in the NAFLD patients. All NAFLD patients were not cirrhosis. The number of words during VFT was significantly higher in the healthy controls compared to the NAFLD patients.

**Table 1:** Characteristics of NAFLD patients and healthy controls.

Characteristic	NAFLD (n = 24)	Controls (n = 15)	p- value
Age (years)	54 (47–61)	51 (48–54)	0.203
CES-D (score)	4 (2–9)	4 (1–6)	0.486
PSQI (score)	5 (3–6)	5 (2–7)	0.853
number of words during VFT	12 (10–14)	14 (13–17)	0.032*
Body mass index (kg/m^2^)	28.9 (25.8–32.1)	20.1 (18.6–21.8)	< 0.001*
ALT (U/L)	49 (29–62)	-	-
ALP (U/L)	264 (210–298)	-	-
Total bilirubin (mg/dl)	0.7 (0.6–1.0)	-	-
LDL-C (mg/dl)	108 (91–131)	-	-
HDL-C (mg/dl)	51 (46–61)	-	-
Triglyceride (mg/dL)	122 (81–139)	-	-
Ferritin (ng/dL)	114 (62–223)	-	-
Fasting plasma glucose (mg/dl)	109 (97–129)	-	-
HbA1c (%)	6.0 (5.5–6.5)	-	-
FIB4	1.27 (0.85–1.93)	-	-
Diabetes mellitus (cases)	10		
Dyslipidemia (cases)	14	-	-
Hypertension (cases)	7	-	-

Data are means median (interquartile range).

Statistical analysis was conducted using Mann-Whitney *U* test.

*Significant differences between NAFLD and control group (*p* < 0.05).

CES-D, Center for Epidemiologic Studies Depression Scale; PSQI, Pittsburgh Sleep Quality Index; VFT, verbal fluency task; ALT, alanine aminotransferase; ALP, alkaline phosphatase; LDL-C, low-density lipoprotein cholesterol; HDL-C, high-density lipoprotein cholesterol.

**Fig 2:**
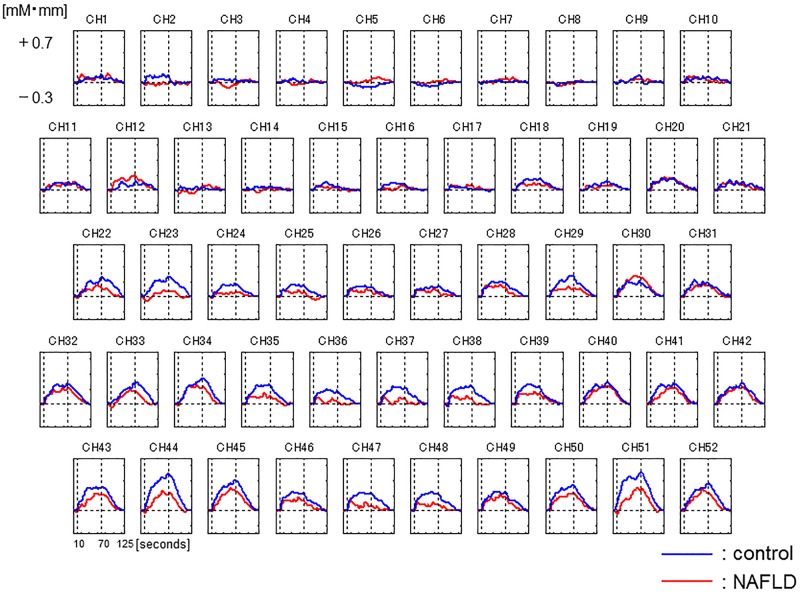
Time-dependent changes in oxygenated hemoglobin concentration in response to a verbal fluency task in NAFLD patients (red) and healthy controls (blue).

**Fig 3:**
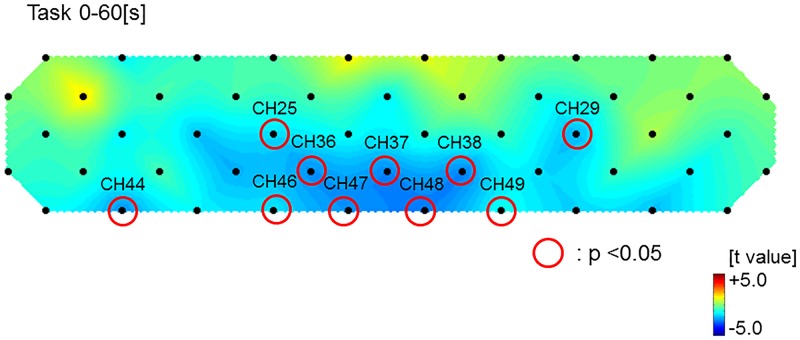
Topographic images of the differences in mean of oxygenated hemoglobin changes between NAFLD patients and healthy controls. Red circles indicate the channels that were significantly smaller in NAFLD patients compared to healthy controls.

**Fig 4:**
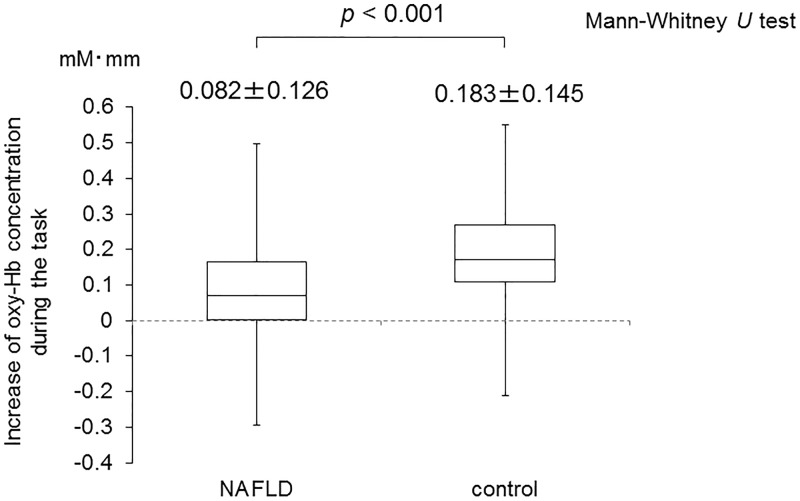
Comparison of the mean of oxygenated hemoglobin concentration in frontal channels (ch 36–38, ch 46–49) during the 0-60-s task period between NAFLD patients (n = 24) and healthy controls (n = 15).

The ground average oxy-Hb concentration during VFT is shown in Fig 2. The oxy-Hb concentrations were lower in the NAFLD patients in many channels than in the healthy controls. Fig 3 shows a topographic image of the difference in mean oxy-Hb concentration during VFT 0–60 s between the NAFLD patients and healthy controls. The red, green and blue colors indicate increase, no change, and decrease of average oxy-Hb concentration, respectively, in the NAFLD patients compared to the healthy controls. Each mean oxy-Hb concentration of the ten channels (Ch 25, 29, 36–38, 44, 46–49) was significantly lower in the NAFLD patients than in the healthy controls, respectively. Moreover, the mean value of oxy-Hb during the 60-s task periods in seven channels (Ch 36–38, 46–49) was significantly lower in the NAFLD patients compared to the healthy controls (0.082 ± 0.126 vs. 0.183 ± 0.145, *P* < 0.001) (Fig 4). On the other hand, there were no significant changes in channels of temporo-parietal areas. In the NAFLD patients, there were no significant correlations between mean oxy-Hb concentration, laboratory findings, and BMI (S1 Table).

## Discussion

In the present study, brain activity of female NAFLD patients was evaluated by NIRS. NIRS showed that an increase in cerebral oxy-Hb concentration in response to the VFT was small in the NAFLD patients compared to the healthy controls in the frontal lobe despite no significant difference in the CES-score between the two groups. To the best of our knowledge, our study appears to be the first to evaluate NAFLD patients using NIRS.

NIRS is one of the imaging tools for evaluating brain activity [12]. A positive correlation has been confirmed between oxy-Hb concentration by NIRS and blood-oxygen-level-dependent signaling by fMRI [19–20]. Therefore, NIRS has been applied to objective evaluation of depressive state in psychiatric disorders [13–15]. With respect to liver disease, impaired brain activity in cirrhotic patients with minimal hepatic encephalopathy has been confirmed by NIRS [21]. We recently reported reduced frontal activation in chronic hepatitis C patients with interferon-based therapy using NIRS [18]. Other than these studies mentioned above, there have been no reports on evaluation using NIRS in patients having NAFLD with other metabolic diseases such as hypertension, dyslipidemia and diabetes mellitus.

NAFLD is the hepatic form of metabolic syndrome; therefore, many lifestyle factors can affect NAFLD [22]. We recently showed that sleep shortage tends to be associated with NAFLD in Japanese women [23]. Other investigators have shown that depression is also affected by many lifestyle factors such as eating, exercise, and sleeping [24]. Consequently, previous studies have indicated an association between depression and NAFLD including histological features [1–4]. Interestingly, we found a decrease in oxy-Hb concentration during tasks in NAFLD patients despite having CES-D scores similar to those of healthy controls. This result may reflect a decrease in brain activity in NAFLD before the patients are experiencing depression.

We confirmed the decreased number of words during VFT in the NAFLD patients in addition to decreased oxy-Hb concentration in present study. This finding can indicate decreased oxy-Hb concentration reflects a decrease in brain activity in NAFLD patients because there is generally a positive correlation between brain activity and numbers of words articulated in VFT [25–26]. Lower cognitive performance in patients with NAFLD has been recently reported by epidemiological study [27]. Therefore, decline of cognition function may explain the decrease in number of words and oxy-Hb concentration during VFT in NAFLD patients. Moreover, sleep shortage has been reported to decrease the NIRS signal [28]. Short sleep duration and poor sleep quality have been reported in NAFLD patients [23, 29]; thus, sleep disorder may be a reason for decreased oxy-Hb concentration during VFT. However, decreased oxy-Hb concentration during VFT in NAFLD cannot be explained only by sleep disorder because there was no significant difference in the PSQI score, including the sleep duration component, between the two groups in the present study.

Although there was not significant, the mean of oxy-Hb concentration around right temporal area (CH12) tended to be higher in patients with NAFLD compared to that of healthy controls. Previous study reported satiation decreased regional cerebral blood flow in several regions including temporal cortex [30]. NAFLD patients were fasting at the time of NIRS, other, healthy controls were almost after meal. Therefore, satiation in healthy control may explain the higher oxy-Hb concentration around right temporal-parietal area in in patients with NAFLD.

The present study had several limitations. First, there have been some concerns regarding evaluation by NIRS [31–32]. NIRS signals during VFT may be influenced by skin blood flow. Furthermore, metabolic complications or medications also may affect NIRS signals. Evaluation by NIRS for patients with metabolic complications will solve the problems in the future. Secondly, the sample size was small and the subjects were limited to only female NAFLD patients. Moreover, some NIRS signals of temporal area couldn’t detect in all subjects because of artifacts, therefore, it can’t deny the possibility of significance differences in those channels. Other, there was no liver histological evaluation of the NAFLD patients. Unfortunately, there was no correlation between average oxy-Hb concentration and laboratory findings in the present study. This may result from the small sample size or that the NAFLD patients in the present study were well-controlled by treatment. In the future, a large-scale study including male patients and histological evaluation is required to elucidate the effect of brain activity in NAFLD.

## Conclusions

Decreased brain activity may be associated with female NAFLD patients without depression. NIRS can be a useful tool for the evaluation of brain activity in NAFLD patients.

## Supporting information

S1 Table Correlations between mean oxy-Hb concentration in frontal channels (Ch 36–38, 46–49), laboratory findings, and Body mass index. (DOCX)

S1 File Level data of subjects. (PDF)
