# Supplementary material for: Reduced brain activity in female patients with non-alcoholic fatty liver disease as measured by near-infrared spectroscopy
Source: PLoS One. 2017 Apr 4;12(4):e0174169. doi: 10.1371/journal.pone.0174169 (PMC5380307; doi:10.1371/journal.pone.0174169)
Supplement: S1 Table — (DOCX) [file pone.0174169.s001.docx]

|  | coefﬁcient of correlation [r] | *p*-value |
| --- | --- | --- |
| Body mass index (kg/m^2^) | -0.030 | 0.884 |
| ALT (U/L) | 0.192 | 0.357 |
| ALP (U/L) | 0.234 | 0.262 |
| Total bilirubin (mg/dl) | 0.028 | 0.948 |
| LDL-C (mg/dl) | 0.031 | 0.885 |
| HDL-C (mg/dl) | 0.249 | 0.249 |
| Triglyceride (mg/dL) | -0.034 | 0.869 |
| Ferritin (ng/dL) | 0.199 | 0.412 |
| Fasting plasma glucose (mg/dl) | -0.119 | 0.573 |
| HbA1c (%) | -0.091 | 0.673 |

**S1 table.**

**Correlations between mean oxy-Hb concentration in frontal channels (Ch 36-38, 46-49), laboratory findings, and Body mass index**

Correlations between variables were measured using Spearman’s rank correlations. ALT, alanine aminotransferase; ALP, alkaline phosphatase; LDL-C, low-density lipoprotein cholesterol; HDL-C, high-density lipoprotein cholesterol.
