# Supplementary material for: Reduced brain activity in female patients with non-alcoholic fatty liver disease as measured by near-infrared spectroscopy
Source: PLoS One. 2017 Apr 4;12(4):e0174169. doi: 10.1371/journal.pone.0174169 (PMC5380307; doi:10.1371/journal.pone.0174169)
Supplement: S1 File — (PDF) [file pone.0174169.s002.pdf]

	age	sex	BMI	CES-D sc	VFT	PSQIG	ALT	ALP	TB
NAFLD 01	59	F	32.5	1	12	1	132	460	1
NAFLD 02	53	F	30.7	14	10	10	119	259	0.5
NAFLD 03	70	F	26.9	5	9	4	14	411	0.6
NAFLD 04	66	F	24.8	5	12	5	95	228	0.7
NAFLD 05	63	F	32	3	8	0	60	325	1.4
NAFLD 06	73	F	26	2	8	6	53	330	0.7
NAFLD 07	53	F	32.3	14	12	9	33	258	0.6
NAFLD 08	61	F	29.3	1	12	3	38	268	1.4
NAFLD 09	48	F	32.7	2	25	6	63	231	0.7
NAFLD 10	46	F	29.3	13	10		87	211	0.7
NAFLD 11	60	F	25.9	4	11	13	34	144	0.7
NAFLD 12	43	F	28.7	0	12	1	30	153	0.1
NAFLD 13	50	F	22.1	15	9	7	27	299	0.5
NAFLD 14	40	F	23.7	3	10	2	17	207	1
NAFLD 15	40	F	29.1	9	13	5	115	269	0.9
NAFLD 16	52	F	28.1	3	10	6	46	290	0.1
NAFLD 17	47	F	32.3	24	21	6	41	169	0.6
NAFLD 18	43	F	34	3	15	6	52	296	1.9
NAFLD 19	62	F	28.6	9	18	4	59	333	0.7
NAFLD 20	57	F	25.4	0	7	2	27	297	0.8
NAFLD 21	61	F	22.8	7	14	6	53	295	1.3
NAFLD 22	63	F	24.3	0	11	5	24	157	1
NAFLD 23	55	F	32	0	16	3	24	238	0.9
NAFLD 24	36	F	32.4	9	17		61	167	0.7
NC01	59	F	22.6	6	13	9			
NC02	57	F		0	12				
NC03	42	F	21.9	5	14	9			
NC04	55	F	26.6	17	14	9			
NC05	50	F	16.8	2	12	4			
NC06	47	F	18.5	6	15	6			
NC07	48	F	20.1	3	23	6			
NC08	44	F	20.3	4	16	1			
NC09	56	F	18.8	0	15	1			
NC10	52	F	20.1	0	21	1			
NC11	47	F	18.3	1	14	2			
NC12	51	F	21.4	6	27	6			

NC13	51	F	27	0	7	2
NC14	51	F	18.5	12	11	7
NC15	51	F	20	4	18	2

NIRS

NAFLD

Type:1 Hb:Oxy

Pre 10s:

Name	ch1	ch2	ch3	ch4	ch5	ch6	ch7	ch8	ch9
NAFLD 01	-0.000726	-2.7E-05	.	-0.00263	-0.000263	-0.00094	-0.00197	-0.00172	-0.00129
NAFLD 02	0.001313	0.001015	.	-2.3E-05	-0.00022	0.004031	0.002148	0.001864	0.000506
NAFLD 03	-0.002383	-0.00439	.	-0.00038	-0.000465	-0.00124	-0.00122	-0.00159	-0.00095
NAFLD 04	0.004017	0.001679	0.002181	0.000661	0.001303	-0.00038	-0.00211	-0.00044	0.001395
NAFLD 05	0.001635	-0.00134	.	0.001336	0.000644	-0.00143	-0.00155	0.000898	0.000706
NAFLD 06	0.001014	-0.00041	0.001531	-1.6E-05	0.000154	0.000652	0.000732	0.0008	0.003224
NAFLD 07	-0.002566	-7.8E-05	-0.00257	0.000771	-0.000022	0.000126	.	0.000234	.
NAFLD 08	.	0.00019	0.000568	-0.00087	0.000033	-0.00256	-0.02174	-0.00198	-0.00151
NAFLD 09	-0.000154	-0.00392	0.002257	-0.00158	-0.002469	0.000018	-0.00239	0.002151	-0.00058
NAFLD 10	.	-0.03163	.	.	-0.004607	0.002582	-0.01273	-0.00871	-0.00217
NAFLD 11	.	.	-0.00347	-0.00066	-0.000196	-0.00093	-9.1E-05	-0.00042	0.00313
NAFLD 12	.	.	0.000983	0.000477	-0.001679	-0.00085	-0.00226	-0.00089	-0.0012
NAFLD 13	.	.	.	.	.	0.001746	.	.	.
NAFLD 14	-0.003378	-0.00561	-0.00211	0.000764	-0.00073	-0.00414	-0.01013	-0.0011	-0.0069
NAFLD 15	-0.001265	0.003883	0.01889	0.006686	0.002627	0.000741	-0.00038	0.001004	0.00406
NAFLD 16	.	.	-0.00165	0.002049	.	.	.	.	.
NAFLD 17	-0.000689	-0.00236	-0.00071	-6.6E-05	0.000262	0.000095	0.002141	0.000722	-0.00103
NAFLD 18	-0.001413	-0.00257	-0.00074	-0.00071	-0.001286	-0.0014	-0.0005	.	.
NAFLD 19	-0.004595	-4.8E-05	-0.00111	-0.00195	-0.00286	-0.00279	-0.00127	-0.00102	-0.00149
NAFLD 20	.	.	.	.	.	.	0.001021	.	0.003935
NAFLD 21	0.000313	0.002479	0.004931	0.003209	0.002223	0.002399	0.006477	0.005098	0.004268
NAFLD 22	.	.	.	0.003304	.	.	0.001231	-0.00207	.
NAFLD 23	.	.	.	-0.00287	-0.001578	-0.00127	-0.0032	-0.00053	-0.00022
NAFLD 24	-0.001363	.	0.000878	-0.00254	-0.002503	-0.00375	-0.00373	-0.00262	-0.00295

Task1 60.0s

Name	ch1	ch2	ch3	ch4	ch5	ch6	ch7	ch8	ch9
NAFLD 01	-0.029272	-0.17545	.	0.034567	-0.024298	0.020919	-0.09428	-0.2079	-0.02559

NAFLD 02	0.027381	0.024268	.	0.059658	0.183475	0.268799	0.247467	0.11215	0.223794
NAFLD 03	0.16985	0.16738	.	0.305743	0.213766	0.001179	−0.00076	0.032442	0.10454
NAFLD 04	0.220065	0.078876	0.085581	0.102426	0.120693	0.069746	−0.04433	0.04963	0.095914
NAFLD 05	0.480924	−0.05501	.	0.027356	0.049163	−0.18175	0.069187	0.30011	0.26125
NAFLD 06	0.013487	0.032559	0.077507	0.064032	0.028193	0.021591	0.019577	0.050392	0.025028
NAFLD 07	0.180756	−0.52045	−0.82438	−0.35769	−0.131696	−0.1751	.	−0.13532	.
NAFLD 08	.	−0.04875	−0.18853	−0.29708	−0.111642	−0.102	−0.04202	−0.0746	0.053175
NAFLD 09	0.21174	0.16206	0.313319	0.005429	−0.025954	0.112265	−0.16088	−0.17249	−0.21949
NAFLD 10	.	−0.17088	.	.	0.011846	−0.06046	−0.01085	−0.01238	0.111281
NAFLD 11	.	.	−0.13786	0.07527	0.054903	0.05989	0.056742	0.070518	0.193933
NAFLD 12	.	.	−0.11149	0.127885	0.014588	0.027153	0.040875	−0.17644	−0.08197
NAFLD 13	.	.	.	.	.	−0.16049	.	.	.
NAFLD 14	0.213545	0.059676	0.016636	−0.09491	−0.118218	−0.03883	−0.00031	0.044827	−0.16699
NAFLD 15	0.094238	−0.00307	−0.2428	−0.34814	−0.103246	−0.05083	−0.03306	0.004967	0.004457
NAFLD 16	.	.	0.11311	0.250129	.	.	.	.	.
NAFLD 17	−0.116402	−0.00979	0.043118	0.073256	0.040373	0.037244	−0.04133	−0.04909	−0.1127
NAFLD 18	0.114508	0.178123	0.232141	0.097024	0.079525	0.122224	0.163857	.	.
NAFLD 19	−0.14564	−0.15347	−0.05534	−0.0049	0.020615	−0.02967	−0.04874	−0.02449	−0.0531
NAFLD 20	.	.	.	.	.	.	0.215703	.	0.236534
NAFLD 21	−0.032681	0.103767	0.301356	0.137799	0.097456	0.040465	0.00898	0.040331	−0.05293
NAFLD 22	.	.	.	−0.00597	.	.	0.085391	−0.1089	.
NAFLD 23	.	.	.	−0.27306	−0.220691	−0.01283	0.027673	−0.11678	−0.19467
NAFLD 24	−0.544077	.	−0.08889	−0.18293	−0.079341	−0.06343	−0.0863	−0.05716	0.022396

Healthy control

Type:2 Hb:Oxy

Pre 10s:

Name	ch1	ch2	ch3	ch4	ch5	ch6	ch7	ch8	ch9
NC01	−0.003397	0.002743	0.001134	0.003496	0.00165	0.000615	−0.00181	.	.
NC02	−0.000595	0.00158	−0.00024	0.001441	0.000336	0.000605	0.000221	−0.00074	.
NC03	0.001001	.	.	0.002524	0.002524	0.00031	.	0.003458	.
NC04	0.000295	−0.00117	.	−0.00303	.	.	−0.00202	−0.00203	.
NC05	−0.007157	−0.00409	−0.00252	−0.00105	−0.001347	−0.00274	−0.00541	−0.00112	.
NC06	0.000428	−0.00765	0.000341	.	0.002072	0.000545	−0.00058	0.00061	.
NC07	−0.008922	−0.0006	−0.00041	−0.00029	−0.000269	0.00058	0.000386	−0.00135	−5.6E−05
NC08	−0.001523	.	−0.00117	−0.00544	−0.000018	−0.00054	−0.00197	−0.00269	−0.00461
NC09	0.000071	−0.00159	0.000694	−0.00047	−0.001344	−0.00076	−0.00054	.	−0.00231
NC10	−0.004298	−0.00287	0.000095	0.000127	−0.000141	−0.00057	−0.00014	0.002198	0.001236

	ch1	ch2	ch3	ch4	ch5	ch6	ch7	ch8	ch9
NC11	0.002486	0.007258	-0.00162	-0.00452	-0.001692	-0.0009	-0.00195	-0.00178	-0.00408
NC12	-0.000978	-0.00101	-0.00158	-0.00169	0.000464	0.000237	-0.00187	-0.00013	-0.00023
NC13	0.001491	0.001074	0.000981	0.003658	0.001398	0.001826	0.011059	0.006596	0.005246
NC14	-0.00101	0.000223	0.002581	-0.00118	-0.002969	-0.00235	-0.00193	-0.00036	-0.00115
NC15	-0.001385	-0.00109	-1.1E-05	0.006363	0.00396	0.004199	.	.	-0.00693

Task1 60.0s:

Name		ch1	ch2	ch3	ch4	ch5	ch6	ch7	ch8	ch9
	NC01	0.00117	0.047493	0.057432	0.182237	0.121383	0.06654	0.10771	.	.
	NC02	-0.089505	-0.18873	-0.08851	0.090762	-0.109699	-0.05526	0.0558	0.008045	.
	NC03	-0.072516	.	.	0.254787	-0.238682	-0.40981	.	-0.48917	.
	NC04	0.030057	-0.04798	.	0.216294	.	.	0.105469	0.062381	.
	NC05	0.112425	0.226301	0.168208	0.078121	0.068065	0.066671	0.065436	0.096521	.
	NC06	0.316871	0.269009	0.158748	.	-0.202053	0.092235	0.055283	0.088851	.
	NC07	0.000426	-0.11366	-0.07673	0.009436	0.021797	-0.00032	0.01943	0.00306	0.031717
	NC08	0.056666	.	-0.14882	-0.27533	-0.229747	-0.04917	-0.09737	0.00618	-0.09258
	NC09	0.271504	0.539786	0.057708	-0.45329	0.14096	-0.01368	-0.08336	.	0.47824
	NC10	-0.059481	-0.09343	0.049516	0.034622	-0.04005	-0.07056	0.001621	0.034028	-0.0514
	NC11	0.237091	0.323498	0.108107	0.133289	-0.010297	-0.01569	0.038857	0.068034	0.137958
	NC12	-0.108649	-0.10333	-0.05504	-0.02343	-0.058513	-0.05254	0.065051	0.006047	-0.00488
	NC13	0.0721	0.069879	0.129494	0.080097	-0.005207	-0.03014	-0.11275	-0.03007	-0.07444
	NC14	0.01248	0.057717	0.141145	0.023626	-0.030556	-0.03597	-0.09319	0.003186	0.042984
	NC15	-0.045524	-0.04435	-0.07835	-0.05999	-0.020763	-0.02725	.	.	-0.18936

TG	HDL	LDL	Ferrtin	glu	A1c	FIB4
98	69	121	227	135	6.4	2.04
125	43	82	183	204		2.78
77		95		96	5.5	3.28
120	48	131	223	141		
137	58	118	169	96	6.5	1.82
136	52	164		114	5.9	3.11
180	59	80	71	94	5.4	0.98
49	54	177	351	75	5.2	1.31
140	49	131		120	6.1	1.01
112	76	107	266	89	5.6	1
74	64	131	174	127	6.8	1.27
146	28	97	24	107	5.7	0.5
82	61	171	62	102	6	0.71
233	60	92	56	97	5.5	0.44
158	33	66	39	130	8.5	0.68
99	51	89	<10	100	5.2	1.68
113	50	172		157	9.3	0.72
39	45	66	86	107	5.5	2.3
139	66	122	237	133	7.4	1.22
139	44	109	97	117	6.4	1.1
65	62	103				2.56
123	47	100		114	6.1	1.42
158	48	160	114	109	6.7	1.33
48	42	87	51	96	5.2	0.6

ch10	ch11	ch12	ch13	ch14	ch15	ch16	ch17	ch18	ch19	ch20	ch21	ch22	ch23
-0.00165	-0.00331	-0.00387	.	-0.00642	0.000961	-0.00213	-0.00125	-0.00211	-0.00292	-0.00264	-0.00126	-0.00474	-0.00352
-0.00051	-0.0014	0.002669	.	.	.	0.002002	0.001988	0.000515	.	-0.00071	-0.00152	0.001015	-0.00027
0.00203	0.006218	-0.00069	.	-0.00547	-0.00076	0.000512	-0.0001	-0.00126	-0.00289	-0.00285	0.00119	-0.01465	0.012843
0.001122	0.003006	0.005403	0.002889	0.002084	0.002216	0.000992	0.001563	-0.00091	0.001601	0.000823	0.001319	0.002072	.
-0.00144	0.00302	-0.00147	-0.00235	-0.00324	0.001441	-0.00094	-0.00013	-0.00052	0.001089	0.000456	0.000348	0.000625	-0.00055
-0.00064	0.000517	-0.00145	0.000303	0.000655	0.00091	0.00053	0.000649	0.000993	0.000243	0.001175	0.000218	0.000155	0.000513
-0.00075	-0.00188	-0.00216	-0.00301	-0.00197	-0.00176	-0.00176	-0.00236	-0.00085	.	-0.00112	-0.00199	-0.0009	-0.0023
.	-0.00135	.	0.001336	.	-0.00108	-0.0001	0.002317	-0.00606	-0.002	.	-0.00091	-0.00268	-0.0061
0.000733	-0.00428	.	.	0.000692	0.002652	-0.00146	-0.01214	0.003542	0.000859	0.00238	0.000629	0.000124	0.004113
.	.	.	-0.02681	.	-0.01103	-0.01114	-0.0044	-0.00227	-0.00078	-0.00033	-0.002	-0.00182	-0.00258
0.002678	-0.00137	-0.00354	.	-0.00205	-0.00093	-0.00093	-0.00016	-0.00046	0.002085	0.003442	0.002221	-0.001	.
.	.	.	.	-0.00131	0.000054	-0.00074	-0.00158	-0.00067	0.00033	-0.00244	.	0.003275	0.002238
.	.	.	.	-0.00232	.	.	.	.	.	.	.	-0.00392	-0.00237
.	-0.00309	-0.00318	-0.00292	0.000306	0.00497	-0.00023	-0.00433	-0.00265	.	-0.00361	.	.	.
0.000902	-0.00109	-0.00285	-0.00095	0.006061	-0.00251	0.001356	-0.00396	0.000614	-0.00507	-0.00619	-0.00771	-0.00212	0.00144
.	0.004013	-0.00057	-0.00825	-0.00101	0.001572	-6.2E-05	0.001075	.	-0.00337	.	.	.	.
-0.00292	-0.00199	0.000571	-0.00103	-0.00021	0.000823	-0.00068	-0.00075	-0.00039	0.000998	-0.00248	-0.00289	-0.00055	.
.	.	.	0.00148	0.002556	0.001153	-0.0005	-0.00389	.	.	-0.00019	.	.	.
-0.00102	-0.00247	0.000792	0.000086	-0.00414	-0.00381	-0.004	-0.00337	-0.0027	-0.00174	-0.00125	-0.00078	0.002844	0.00057
.	.	.	.	0.004484	0.001669	0.000946	0.000431	.	0.001764	.	.	.	.
0.001607	-0.00309	0.012945	0.008628	0.00124	0.001871	0.000092	0.001322	0.005931	0.005951	-0.00165	-0.00232	0.01255	.
.	-0.0005	.	.	.	0.010462	.	.	0.004735	0.000116	-0.00242	-0.00094	.	.
-0.00221	.	.	.	-0.00121	-0.0018	0.001492	-0.00138	-0.00177	0.000887	0.000044	0.000558	.	-0.0002
-0.00518	-0.0016	.	0.000666	-0.00109	-8.6E-05	-0.00037	-0.00244	-0.00151	-0.00356	-0.00187	-0.00404	-0.00013	-0.00092

ch10	ch11	ch12	ch13	ch14	ch15	ch16	ch17	ch18	ch19	ch20	ch21	ch22	ch23
0.090623	0.442458	0.061336	.	0.063143	0.007431	0.034529	-0.03285	0.1099	-0.02285	-0.06329	0.105399	0.299343	0.166895

0.163139	0.161164	0.248252	.	.	.	0.094956	0.140334	0.137034	.	0.220013	0.217399	0.421256	0.469681
0.094145	0.081561	0.221286	.	0.477591	0.258013	0.329084	0.137615	0.159064	0.237034	0.19452	0.151198	0.104794	0.426133
0.195593	0.135523	0.282964	0.141015	0.12793	0.169496	0.163053	0.151902	0.134627	0.248213	0.394626	0.255366	0.198344	.
0.108504	0.300968	0.23696	0.03137	-0.00226	0.084469	0.061498	0.179761	0.14938	0.460587	0.228941	0.255536	0.447042	0.276601
0.023525	0.020467	0.025334	0.024485	0.050878	0.022169	0.043507	0.031884	0.06566	0.045301	0.029339	0.039255	-0.01818	0.026553
0.209908	0.107593	-0.10071	-0.82563	-0.3759	0.318116	-0.05579	0.124619	-0.06358	.	0.263881	0.284979	0.159743	0.022429
.	-0.22622	.	-0.13594	.	-0.20438	-0.08732	-0.01766	-0.01327	-0.03674	.	0.014563	-0.20608	-0.39786
0.217008	0.011182	.	.	-0.05741	0.029912	-0.04496	0.105041	0.32664	-0.1422	0.00859	0.080787	0.072566	0.062167
.	.	.	0.0637	.	-0.01006	0.036994	-0.05401	0.055323	0.087503	0.294576	-0.04685	-0.3374	-0.20458
0.154517	0.158863	0.150286	.	-0.05394	0.085629	0.120546	0.111375	0.073213	-0.03334	0.288958	0.184496	0.206536	.
.	.	.	.	-0.07027	0.119387	-0.04152	0.087334	0.072597	-0.17218	-0.05118	.	0.037155	-0.0521
.	.	.	.	0.090601	.	.	.	.	.	.	.	0.202232	0.039665
.	0.216529	0.276361	0.17787	0.032775	-0.01587	-0.04499	-0.01204	0.09124	.	0.009497	.	.	.
-0.0763	0.059984	0.221633	-0.0737	-0.12319	-0.22199	-0.08421	0.017177	-0.06559	0.01831	0.14765	0.003593	0.110633	0.118727
.	0.013266	0.269596	0.196024	0.136957	0.133505	0.026529	0.022483	.	-0.06085	.	.	.	.
-0.17033	0.124565	-0.00791	0.136803	0.024831	0.032404	0.051646	0.094466	-0.06573	-0.07618	0.015496	-0.05431	0.187701	.
.	.	.	0.224586	0.020474	0.133968	0.035392	0.102766	.	.	0.180543	.	.	.
-0.05487	-0.0726	-0.15604	-0.17807	-0.10286	-0.05452	-0.05718	-0.03499	-0.10833	-0.05025	-0.0567	-0.22244	-0.12797	-0.13862
.	.	.	.	-0.04918	-0.11514	-0.24555	-0.18239	.	0.14969	.	.	.	.
-0.03849	0.016783	0.068524	0.091817	0.112516	0.068306	0.07759	0.052394	0.050905	0.095756	0.013846	0.027219	-0.01803	.
.	-0.283	.	.	.	0.110793	.	.	0.295242	0.075024	0.011881	0.015497	.	.
-0.10012	.	.	.	-0.11355	-0.08873	0.096873	-0.03134	-0.02592	-0.09986	0.262809	0.144457	.	-0.29553
-0.02289	-0.26215	.	-0.13651	-0.28364	-0.07633	-0.0383	0.009844	-0.02668	-0.03403	0.036852	-0.04979	-0.21046	-0.19143

ch10	ch11	ch12	ch13	ch14	ch15	ch16	ch17	ch18	ch19	ch20	ch21	ch22	ch23
-0.00159	-0.00125	0.001985	.	0.001496	0.00127	0.002779	0.002087	-0.00368	.	-0.00042	-0.00031	-0.00107	-0.00157
.	-0.00225	0.007045	0.000216	0.000433	-0.00276	-0.00016	0.000192	-8.9E-05	-0.00064	0.001702	-0.02023	0.001284	0.005643
0.006649	0.004099	.	0.000086	.	0.004115	0.003359	0.005741	.	0.008612	0.009751	0.003512	0.007382	0.011322
-0.00138	0.000833	-0.00035	.	-0.00086	.	-0.00043	-0.00114	-0.00034	-0.00115	-0.00013	0.000079	-0.00267	.
.	-0.00206	.	-0.0028	-0.00085	0.000496	-0.00025	-0.00052	-0.00063	-3.4E-05	.	-0.00282	.	.
-0.0025	-0.00012	.	0.001068	0.000819	.	0.0011	0.002223	0.000352	-0.00056	0.000752	-0.00131	.	.
-0.00445	0.00414	.	-0.00087	-0.00249	0.00148	0.000709	-0.00025	0.000552	-0.00113	0.001979	0.000378	.	.
-0.00439	-0.00187	-0.00088	-0.00465	-0.00289	.	-0.00106	-0.00063	-0.00298	-0.00407	0.005038	-0.00041	-0.00404	.
-0.00169	0.000218	0.001345	0.002265	0.001258	-0.00183	-0.00361	-0.0007	-0.00158	.	0.000547	-0.00151	0.001886	0.002737
-0.00142	-0.00432	-0.00135	0.000181	0.000881	0.000501	0.000097	0.001365	-0.00065	0.004667	0.003468	-0.00253	0.002226	0.002805

-0.00117	-0.00074	0.001656	0.006235	0.001937	0.000524	0.000452	-0.00199	0.00106	-0.0007	-0.00399	-0.00147	0.00375	0.004719
.	-0.00285	-0.00195	-7.7E-05	0.000308	-0.00083	0.000578	-0.0007	-0.00191	0.001548	-0.00123	.	-0.00037	0.000044
.	0.000314	0.001789	0.002298	0.003652	0.002274	0.002721	0.002172	0.002927	0.006412	0.00125	.	0.002019	0.004114
-0.00083	-0.00101	-0.00193	0.00094	0.000406	-0.0018	0.000347	-0.00233	-0.00033	-0.00298	0.000687	-0.00087	0.000395	-0.00199
-0.00337	-0.00099	0.00184	-0.00361	0.002469	0.000343	0.001355	-0.00077	.	-0.0037	-0.00185	-0.0038	.	-0.00303

ch10	ch11	ch12	ch13	ch14	ch15	ch16	ch17	ch18	ch19	ch20	ch21	ch22	ch23
0.261924	0.089214	0.077016	.	0.182664	0.185277	0.127524	0.046208	0.283565	.	0.102052	0.206199	0.062086	-0.08532
.	0.017235	-0.09343	-0.09233	-0.06123	-0.11806	-0.02887	0.011514	0.258794	0.235293	0.249048	0.016719	0.224778	0.186271
0.060434	-0.11126	.	-0.08884	.	0.411822	0.263995	-0.45184	.	-0.19704	0.320373	0.079633	0.380435	0.700643
0.079886	0.255439	-0.0098	.	0.133229	.	0.013201	0.209561	0.21612	0.097775	0.225364	0.039079	0.249095	.
.	0.161169	.	0.188409	0.134755	0.125376	0.209937	0.158828	0.237088	0.306872	.	0.15675	.	.
0.007916	0.148748	.	0.138713	0.164401	.	0.150072	0.07552	0.050806	0.249476	0.076745	-0.02491	.	.
0.15591	0.082546	.	-0.13191	-0.03431	0.071069	0.123304	0.066923	0.111337	0.017956	0.211218	0.21579	.	.
-0.09803	0.054789	0.055946	-0.11897	-0.02954	.	0.068337	-0.04579	-0.02946	-0.00453	-0.12272	0.033515	0.083867	.
-0.06595	-0.0524	0.112518	0.265828	-0.36008	-0.19235	0.055489	0.102366	-0.07875	.	0.156656	0.02693	-0.12916	-0.05558
0.059063	0.091415	-0.04613	-0.04883	0.024295	0.101098	-0.04501	0.141697	0.024642	-0.18193	0.162345	0.000671	0.11699	0.065875
0.167386	0.118518	0.246551	0.102554	0.145117	0.093674	0.06229	0.086917	0.163269	0.339337	0.195931	0.27013	0.226467	0.126161
.	-0.05905	0.02438	-0.02721	0.05092	0.002396	-0.05963	0.064801	0.042633	0.071992	0.076519	.	0.035584	0.20444
.	0.133279	0.072621	0.093666	0.09803	0.029078	0.012271	0.037394	0.133713	-0.0491	0.050539	.	0.159384	0.074007
0.034207	0.024627	0.020769	0.075682	0.07539	0.102259	0.205344	0.007669	0.008441	0.019018	0.02564	0.105409	0.109982	0.226555
-0.10663	-0.05118	0.123003	-0.06017	-0.11202	-0.06441	-0.07083	-0.04326	.	-0.1486	-0.04313	-0.14004	.	0.178511

ch24	ch25	ch26	ch27	ch28	ch29	ch30	ch31	ch32	ch33	ch34	ch35	ch36	ch37
-0.0019	-0.00248	-0.00109	-0.00187	-0.00096	-0.00319	-0.00615	-0.00199	-0.00272	-0.00543	0.004738	-0.00451	-0.0002	-0.00081
.	0.000271	0.004151	0.000126	.	0.002687	0.000419	-0.00036	-0.00021	0.000404	-0.00026	0.00385	0.003869	0.001978
.	0.000232	0.001308	0.00131	0.004779	0.002508	-0.01722	-0.00954	.	.	-0.00173	-0.00128	0.001274	0.000756
0.003773	0.004295	0.001676	0.001774	0.002463	0.002126	0.00004	0.000556	-0.00221	.	.	0.004293	0.002149	-0.00088
-0.00693	.	-1.1E-05	0.00023	0.000929	0.002317	0.001143	0.001193	0.001202	-0.00196	-0.00098	0.002518	0.001634	.
0.000834	0.000998	0.000279	0.000331	-2.8E-05	0.001449	0.002493	0.000617	0.000143	0.001377	0.001212	0.00216	0.000175	0.001661
-0.00444	-0.00332	-0.00303	-0.00176	-0.0036	-0.00187	-0.00306	-0.00194	-0.00364	-0.0024	-0.00086	-0.00405	-0.00283	-0.00353
0.001145	0.001068	0.00169	0.00129	0.001995	0.001222	-0.0025	0.003193	-0.00276	-0.00842	0.001218	-0.0005	0.001442	0.000793
0.003443	0.002302	0.000455	-0.00003	0.001348	0.002053	-0.00278	-0.00013	0.000389	0.000018	0.002526	0.00088	-0.00028	.
-0.00087	-0.00229	-0.00182	-0.00211	-0.00206	-0.00296	-0.00357	0.004615	0.002222	-0.00469	0.002563	-0.00141	-0.00368	0.001288
-0.00067	0.002029	0.000408	0.001189	0.000137	0.003232	.	.	-0.00166	.	.	-0.00015	0.00069	0.001554
-0.00227	-0.0007	-0.00046	-0.00017	-0.00085	-0.00031	-0.00058	0.002274	-0.00213	0.00162	-0.00228	-0.00163	-0.00142	-8.2E-05
-0.00341	-0.0016	0.0029	-0.00016	-4E-06	-0.00182	0.004104	0.000356	-0.00032	0.001736	-0.00174	-0.00263	0.00289	.
-0.00275	0.00088	0.010413	0.004303	-0.00115	-0.00257	0.003817	-0.02712	0.000377	0.003056	0.001524	-0.00073	-0.00148	-0.00046
0.003431	0.002106	0.001329	0.002562	-0.00028	0.00108	-0.00406	-0.0056	.	-0.00402	0.001061	0.001552	0.003298	0.001479
-0.00647	0.000475	-0.00012	0.000506	0.002866	-0.00091	.	.	-0.00094	.	0.004271	.	.	.
0.000042	-0.00063	-0.0012	-0.00065	-0.00103	-0.00014	.	-0.00158	.	.	-0.00294	-0.0009	-0.00212	-0.00085
-0.00062	-0.00037	0.000418	0.000479	-0.00073	.	.	.	-0.00084	.	.	.	0.003837	0.001642
-0.00327	-0.00405	-0.00332	-0.00288	-0.00297	-0.00234	-0.00148	-0.00028	0.00181	0.003248	-0.00041	-0.00621	-0.00719	-0.00273
-0.00084	0.000818	0.001729	0.002617	0.000614	0.000426	.	.	.	-0.00072	.	0.002644	0.001425	0.001986
0.000811	-0.00023	0.000701	-0.00028	-0.00047	0.004164	.	0.006977	.	.	.	0.000886	0.002123	0.002681
-0.00122	.	.	0.000665	0.001336	0.000714	-0.00245	0.000025	.	.	-0.00254	-0.00114	-0.00145	-0.00142
-0.00267	0.00071	0.000582	0.003456	-0.00016	-0.00234	-0.00014	.	.	.	0.000712	-0.00016	0.000047	-0.00092
-0.0012	.	0.002166	0.001677	0.001594	-0.00049	-0.00588	-0.00408	0.000709	-0.0002	-0.00054	0.005057	0.002474	0.002104

ch24	ch25	ch26	ch27	ch28	ch29	ch30	ch31	ch32	ch33	ch34	ch35	ch36	ch37
0.230889	0.078948	0.096636	-0.0527	0.11289	0.091098	0.079966	0.041384	0.223839	0.019163	0.128876	0.109886	0.068816	0.105087

.	-0.03839	0.077966	-0.1191	.	0.255215	0.59764	0.419705	0.654478	0.346786	0.305793	0.062705	0.03088	-0.08926
.	0.32495	0.340448	0.284688	0.263874	0.275876	0.184313	0.136034	.	.	0.237703	0.3818	0.187471	0.22447
0.14092	0.111816	0.170057	0.212435	0.170104	0.177979	0.308101	0.353198	0.064011	.	.	0.16297	0.193794	0.294097
0.083122	.	0.248566	0.192658	0.235548	0.321177	0.237887	0.245355	0.347292	0.264025	0.206739	0.365253	0.317384	.
0.060946	0.039944	0.019877	0.067367	0.068461	0.103002	0.068289	0.034424	0.012882	0.02861	0.045083	0.135254	0.025987	0.070385
-0.16693	0.354557	0.327023	0.183751	0.756733	0.209316	0.224998	0.229107	0.102409	0.161747	0.269248	0.111057	0.158566	0.124057
-0.15005	-0.13508	-0.06326	-0.04598	0.053348	-0.03429	-0.04509	-0.06769	-0.02538	-0.15064	0.036855	-0.03822	0.082025	0.005787
0.083578	0.078286	0.123516	0.064568	0.193747	0.070019	0.207523	0.090804	0.229832	0.094788	0.11848	0.260049	0.193946	.
0.101994	0.101132	0.044571	0.120399	0.136022	0.027342	0.284043	0.166154	0.004607	-0.34556	0.210841	0.137119	0.119133	0.171448
0.036946	0.140348	0.225971	0.218447	0.161059	0.13227	.	.	0.174356	.	.	0.135766	0.164511	0.256213
-0.07127	0.003642	-0.0101	-0.00927	-0.00321	-0.06581	0.029528	-0.09053	-0.03954	0.024666	0.158003	-0.1082	-0.07224	-0.07366
0.150357	0.057371	0.069569	0.036767	0.076313	0.292623	0.470837	0.123229	0.221929	0.16635	0.351722	0.258394	-0.00437	.
0.146619	0.057614	0.128423	0.084437	0.137177	0.128613	0.320484	0.200669	0.385464	0.48397	0.736082	0.097564	0.084773	-0.00967
0.027809	-0.00946	0.051153	0.033873	0.013852	0.062055	0.283888	0.173786	.	0.114882	0.125264	-0.01339	0.05066	0.086946
0.262631	0.078262	-0.08702	0.078968	0.122096	-0.04775	.	.	0.198424	.	0.267556	.	.	.
0.099194	-0.02061	-0.00794	0.17718	0.067203	-0.1042	.	0.062181	.	.	0.206332	0.007188	-0.13204	-0.1056
0.096368	0.034479	0.045683	0.05556	0.052357	.	.	.	0.044009	.	.	.	0.024194	0.110973
-0.14618	-0.06047	-0.11059	-0.04106	0.006264	-0.11643	-0.04574	-0.05459	0.035827	0.059835	-0.0255	-0.15001	-0.30268	-0.16577
0.059647	-0.02756	-0.08066	-0.02943	-0.07032	0.052849	.	.	.	0.199908	.	0.218977	-0.0423	0.052048
0.091008	0.084732	0.034547	0.031501	0.092449	0.176758	.	0.081249	.	.	.	0.081664	-0.02919	-0.02514
-0.00413	.	.	0.136849	0.20768	0.144989	0.133588	0.10837	.	.	0.059874	-0.1436	-0.0046	0.046974
-0.10439	-0.04842	0.016222	0.077371	-0.05322	-0.10601	0.155934	.	.	.	-0.10235	-0.10399	-0.10887	-0.14225
-0.24555	.	-0.05097	-0.00087	0.054243	-0.00663	-0.18362	-0.09967	-0.14568	-0.10643	-0.05475	-0.0408	-0.07361	-0.02102

ch24	ch25	ch26	ch27	ch28	ch29	ch30	ch31	ch32	ch33	ch34	ch35	ch36	ch37
0.000182	-0.00103	0.000778	0.001845	-0.00083	0.00058	-0.00124	-0.00032	0.001742	-0.00371	.	0.00053	-0.00334	0.003265
-0.00261	-0.00197	-0.0003	-0.00167	-0.00163	-0.0007	.	0.002914	-0.00245	0.00165	-0.0046	-0.00355	-0.00105	-0.00014
0.003346	0.007331	0.005742	0.006028	0.005808	0.005935	0.00033	0.003552	0.010662	.	0.003626	0.007988	0.007566	0.007243
.	.	.	-0.00128	0.000629	0.001678	-0.0015	0.001764	0.000442	0.000389	-0.00121	0.001824	.	-0.00146
-0.00128	-0.00015	0.001514	0.000887	-0.00014	-0.00062	-0.00091	-0.0006	-0.00171	.	0.003982	-0.00121	0.001818	0.002668
0.000175	0.000665	0.000308	-0.00094	0.000763	0.000046	-0.00278	0.001777	-0.00125	.	-0.00084	-0.00032	0.000145	-0.00026
-0.00367	0.001766	0.001918	0.000697	0.000598	0.002046	0.004079	0.001634	0.004729	.	0.003635	0.000704	0.000812	0.001918
-0.00202	-0.00215	-0.00126	0.001711	0.000208	-0.00668	.	-0.00031	-0.00406	-0.00616	-0.0037	-0.00201	-0.00489	-0.00082
-0.00142	-0.00079	-0.00116	0.000236	-0.0009	-0.00063	.	0.006062	0.001321	0.004343	0.003397	-0.00114	-0.0005	-0.00092
0.001438	0.000826	-0.00151	-0.00121	-0.00163	-0.0004	.	.	0.002174	0.008416	0.001765	0.000422	0.001261	0.002124

0.005216	0.001178	0.001805	0.000975	0.001171	0.001992	0.002313	0.005776	0.003298	-0.00134	-0.01355	0.002024	-0.00099	-0.00202
-0.00082	0.000764	0.001091	0.000068	-0.00025	-0.00091	.	-0.00094	-0.00143	0.000588	-0.00355	-0.00329	-0.00095	-0.00114
0.003226	0.002678	0.004131	0.002855	0.003156	0.005489	-0.0076	-0.00001	-0.00016	0.004508	0.001496	0.003057	0.004479	0.005232
-0.0007	0.00018	0.003462	0.002931	0.001271	-0.00125	-0.00048	-1.1E-05	-0.00232	0.00425	.	0.001147	0.00215	.
-8.6E-05	0.002287	0.000923	0.000811	-0.00161	-0.00258	-0.00288	0.001767	.	.	-0.00035	-0.00129	0.000431	0.001355

ch24	ch25	ch26	ch27	ch28	ch29	ch30	ch31	ch32	ch33	ch34	ch35	ch36	ch37
0.132043	0.107062	0.009069	-0.07051	0.181779	0.405783	0.061521	0.063274	0.153702	0.118548	.	0.29213	0.122633	0.215488
0.034522	0.236953	0.044736	0.07288	0.225058	0.309287	.	0.371738	0.142668	0.256416	0.239801	0.272267	0.199775	0.134927
0.167765	0.108753	0.165699	-0.14448	0.193984	0.227501	0.190773	0.100348	0.715116	.	0.423396	-0.03417	0.306969	0.207029
.	.	.	0.182128	0.30668	0.260663	0.113888	0.255852	0.060654	0.122299	0.14609	0.418671	.	0.386612
0.179094	0.173484	0.265755	0.2905	0.30296	0.289079	0.527264	0.4644	0.191513	.	0.166987	0.288476	0.318217	0.553453
0.205051	0.182512	0.202415	0.153313	0.151567	0.131645	0.133081	0.092807	0.141875	.	0.23792	0.217237	0.260039	0.198487
-0.03828	0.218999	0.218867	0.177419	0.279858	0.207559	0.267391	0.040589	0.218678	.	0.418651	0.290518	0.207562	0.188182
0.02594	0.042005	0.104897	0.152838	0.126402	-0.06065	.	0.074477	0.182919	0.129539	0.196418	-0.02389	0.001512	0.128136
0.213244	-0.01832	0.123978	0.237182	0.052495	0.332674	.	0.167663	0.056968	-0.27902	0.22509	0.178551	0.114167	-0.07902
0.061791	0.14178	0.06338	0.046528	0.099059	-0.10106	.	.	0.150124	0.124346	0.087562	0.141383	0.143859	0.185547
0.210986	0.245788	0.179788	0.175037	0.163825	0.270197	0.156071	0.240932	0.067479	0.207606	0.117031	0.211239	0.070779	0.256197
0.225931	0.070331	0.091642	0.064135	0.041816	0.192174	.	0.126988	0.196496	0.202489	0.300831	0.211051	0.110492	0.090423
0.084036	0.114433	0.057452	0.05924	0.131545	0.089709	0.075335	0.129556	0.212174	0.09523	-0.00214	0.193425	0.100215	0.095412
0.146684	0.207127	0.164928	0.248437	0.18242	0.162019	0.092895	0.080413	0.211829	0.47945	.	0.280739	0.264667	.
-0.02771	-0.12567	-0.08104	-0.09608	-0.08585	0.038341	-0.06088	-0.08391	.	.	0.104919	-0.07226	-0.13087	-0.09534

ch38	ch39	ch40	ch41	ch42	ch43	ch44	ch45	ch46	ch47	ch48	ch49	ch50	ch51
-0.00054	-0.00318	0.010582	0.007957	0.001931	.	0.004452	0.007285	-0.00166	-0.00107	-0.00088	-0.00142	0.000918	0.002565
0.00179	.	0.002071	.	-0.00129	.	-0.00149	0.003332	0.001069	0.00105	0.000756	0.002266	0.000994	-0.0011
-0.00017	0.008295	-0.00127	-0.01549	-0.01356	-0.01607	.	-0.0177	0.003847	0.001089	-0.00122	-0.00021	0.004083	.
0.002029	0.000992	0.000851	.	0.002334	.	.	0.003564	0.002265	.	.	0.001429	-9.8E-05	.
0.002124	0.001566	0.001575	0.000872	0.000061	-0.00092	-0.00687	.	0.00198	.	.	0.002706	0.000135	-0.00351
-0.00187	0.001084	0.002234	-0.001	0.000774	0.000475	.	0.002632	0.003221	0.000231	-0.00082	0.002558	0.001299	-0.00025
-0.0021	-0.00269	-0.00451	-0.00302	-0.00216	-0.00302	-0.00315	-0.00124	-0.00163	-0.00429	-0.00217	-0.00219	0.001858	-0.00273
-0.00034	0.002041	0.002413	-0.00631	0.000847	-0.00283	-0.00391	0.000238	-0.0001	0.000131	-4E-06	0.00053	0.001112	-0.00424
0.001831	0.001363	0.001541	0.002309	-0.00421	-0.00036	0.000461	-0.00184	-0.00019	.	.	0.00146	0.000263	0.002082
-0.00248	-0.00076	0.001507	0.000548	0.004661	-0.00141	0.005458	0.000406	-0.00097	-0.00089	-0.00118	-0.00041	-0.0017	.
-0.00067	0.000136	0.001975	.	.	.	.	-0.0027	-0.00182	0.000262	0.000576	-0.00018	0.000178	.
-0.0006	-0.00061	-0.00117	-0.00067	0.000777	-0.00217	-0.00193	-0.00142	-0.00153	-0.00181	-0.00169	-0.00145	-0.00015	-0.00015
0.001087	0.00057	0.003713	0.002327	-0.00338	.	.	-0.00204	0.000117	-0.0007	.	0.001363	0.001482	.
-0.00096	-0.00685	-0.00057	0.002558	.	0.005221	-0.00215	0.00367	0.001047	-0.00086	-0.00345	-0.0015	-0.00086	.
0.006058	-0.00043	0.002322	-0.00211	-0.00107	-0.00657	0.001851	0.003787	0.001793	0.000868	-0.00125	.	.	0.001382
.	.	.	.	-0.00623	-0.00284	.	.	.	-0.00036	-4.1E-05	.	.	.
-0.00163	-0.00209	-0.00349	-0.00345	-0.00263	0.00133	-0.00205	.	-0.0001	-0.0012	-0.0029	-0.00122	-0.00201	.
0.003155	.	.	.	.	.	.	.	.	0.002023	0.00019	0.001495	-0.00026	.
-0.00798	-0.00427	-0.00234	0.003934	-0.00024	-0.00069	-0.00267	-0.00439	-0.00571	0.000229	.	.	-0.00372	-0.00146
0.0011	0.002355	.	.	.	.	.	-0.00129	0.001827	0.000731	0.000653	0.002124	.	.
.	0.001653	.	.	0.012556	.	.	.	0.002148	0.004829	0.000953	-0.00047	0.007013	.
-0.00259	-0.00151	0.000062	0.008988	-0.00555	.	.	-0.00459	-0.00214	0.000867	0.000955	-0.00387	-0.00253	.
0.000264	-0.00176	-0.00015	.	.	0.001257	-0.00273	0.000959	0.000843	-0.00289	-0.00364	0.000372	0.001046	.
0.004285	0.001016	-0.00071	0.001745	-0.00106	0.001914	-0.00123	.	0.005153	.	.	0.00252	0.002236	-0.0041

ch38	ch39	ch40	ch41	ch42	ch43	ch44	ch45	ch46	ch47	ch48	ch49	ch50	ch51
0.053411	0.198482	0.035847	-0.10738	0.12979	.	-0.14839	0.08136	0.158098	0.176512	0.124654	0.090063	0.043582	-0.23183

0.063561	.	0.51546	.	0.759087	.	0.243804	0.29378	0.161995	0.058657	0.021846	0.176738	0.325323	0.419855
0.095938	0.361291	0.317108	0.099412	0.077868	0.299308	.	0.354803	0.382103	0.276673	0.202128	0.151367	0.389458	.
0.226019	0.203282	0.131984	.	0.240856	.	.	0.355412	0.263208	.	.	0.309253	0.250121	.
0.196901	0.244553	0.274979	0.268102	0.215483	0.213472	0.164532	.	0.230567	.	.	0.257842	0.226525	0.336927
0.138778	0.18859	0.086322	0.027313	0.052599	0.0422	.	0.107074	0.123	0.050663	0.096723	0.207331	0.142719	0.044329
0.136407	0.182435	0.277493	0.19972	0.247291	0.116195	0.261642	0.417301	0.163942	0.173004	0.149375	0.252037	0.399584	0.264602
0.041908	0.132723	0.034004	−0.11995	−0.04221	0.038773	0.0241	0.064457	0.049664	−0.00361	−0.0271	−0.00528	0.066259	0.064764
0.293448	0.316065	0.312	0.080377	0.295185	0.108597	0.214546	0.199188	0.307879	.	.	0.381148	0.274439	0.359223
0.088333	0.263051	−0.01893	0.180333	0.125465	−0.04184	0.082353	0.241007	0.135746	0.146658	0.138409	0.187357	0.168114	.
0.18872	0.219949	0.134819	.	.	.	.	0.094219	0.02914	0.181056	0.201791	0.126182	0.169897	.
−0.03424	−0.01618	0.297595	0.228543	0.082135	0.040541	0.086428	0.075245	−0.02721	−0.01528	0.188791	0.010639	0.103515	0.449963
0.014568	0.114881	0.44646	0.300424	0.277886	.	.	0.363834	0.101112	0.395835	.	0.209121	0.281679	.
−0.02319	0.025987	0.290566	0.231631	.	0.502111	0.490025	0.507436	0.151282	0.043821	0.010546	0.039986	0.220045	.
0.086108	−0.06154	0.106158	0.246798	0.065234	0.085513	0.151019	0.131773	0.124175	0.035384	0.112033	.	.	0.186538
.	.	.	.	0.163975	0.121764	.	.	.	0.014003	0.011158	.	.	.
0.045718	−0.01505	−0.08532	0.0125	0.061358	0.30008	0.278409	.	−0.07228	−0.10451	0.007206	−0.11076	−0.08021	.
0.064622	.	.	.	.	.	.	.	.	0.099365	0.052138	0.099989	0.067723	.
−0.13263	−0.08298	−0.07098	0.235199	−0.00289	0.050489	−0.06591	−0.04245	−0.1664	−0.0605	.	.	0.013758	0.059392
0.184077	0.13662	.	.	.	.	.	0.070798	0.281591	−0.07964	0.072491	0.500299	.	.
.	0.15984	.	.	0.175254	.	.	.	0.17378	0.0696	0.088956	0.141481	0.167361	.
0.004672	0.041897	0.104388	0.066144	−0.08937	.	.	−0.21319	0.066022	0.007597	−0.05366	−0.05885	0.034208	.
−0.106	−0.12448	0.008123	.	.	0.25754	0.338698	0.147583	0.013591	−0.00143	−0.02008	−0.05601	0.095049	.
0.061583	0.010122	−0.06653	0.166343	0.026391	−0.14308	−0.13238	.	0.023623	.	.	0.054072	0.030202	−0.16424

ch38	ch39	ch40	ch41	ch42	ch43	ch44	ch45	ch46	ch47	ch48	ch49	ch50	ch51
−0.00072	.	−0.00568	−0.00138	0.00019	−0.00298	.	.	0.000899	−0.00027	0.000167	−0.00029	−0.00112	.
−0.0037	.	−0.00248	−0.00114	−0.00165	−0.00345	−0.00244	−0.00286	−0.00283	−0.00237	−0.00223	−0.00182	−0.00313	−0.00696
.	0.005089	0.003883	0.006225	0.002794	0.015078	0.002501	0.005754	0.008339	0.01035	0.004145	0.005156	0.008972	0.017442
−0.00233	−0.00087	−0.001	−0.00097	−0.00066	−0.00016	−0.00076	−0.00146	−0.00029	0.000159	0.000478	0.000981	0.001048	−0.00194
0.000824	0.00025	0.002802	0.002066	−5E−06	0.004244	.	.	0.001753	0.002653	0.001402	0.000745	0.003407	0.000752
0.000136	0.00087	−4.8E−05	0.000386	−6.8E−05	0.000135	−0.00219	−0.00118	0.000016	0.000069	0.000595	0.000161	0.001127	−0.00041
−0.00615	0.001689	0.000319	0.004301	0.002945	0.005469	.	0.007471	0.001692	−0.00093	−0.00225	0.001258	0.00281	0.009618
−0.00204	−0.0021	−0.00038	−0.00058	−0.00745	−0.00722	−0.00543	−0.00533	−0.00374	−0.0086	−0.00714	−0.00246	.	.
0.000757	−3.6E−05	.	0.00915	0.008743	0.004399	0.004007	0.005889	−0.00073	−0.00086	−4.9E−05	−0.00037	0.004908	0.010962
0.000653	−0.00254	0.002801	0.013002	.	.	.	0.000542	0.001084	0.003695	0.001206	0.000045	0.003791	0.016147

0.000821	0.000974	0.000549	−0.01073	0.002746	0.001827	.	.	0.001443	−0.00323	−0.0032	−0.00094	0.005277	.
−0.00195	−0.00128	−0.00053	−0.00089	−0.00156	0.00388	−0.0021	0.000408	−0.00144	−0.00084	−0.00175	−0.0024	−0.00203	0.000993
0.00357	0.005153	0.001837	0.002741	.	0.003667	.	0.003041	0.008748	0.005984	0.003375	0.004057	0.002165	.
0.001335	0.00257	−0.00138	−0.00106	0.000122	0.004239	0.007374	0.002676	0.003907	.	0.000629	0.002115	0.000208	.
−0.00196	−0.00047	−0.00505	−0.00525	−0.00194	−0.00837	−0.00419	−0.0005	−0.00139	0.005761	0.002313	−0.00156	−0.00082	−0.00661

ch38	ch39	ch40	ch41	ch42	ch43	ch44	ch45	ch46	ch47	ch48	ch49	ch50	ch51
0.139194	.	0.056715	−0.04402	0.099042	0.167902	.	.	0.150302	0.188548	0.108217	0.147061	−0.03084	.
0.223692	.	0.303502	0.248658	0.266148	0.272088	0.379215	0.398151	0.267625	0.107319	0.166053	0.38251	0.478998	0.32429
.	0.572709	0.197289	0.229921	0.244539	0.738505	0.274231	0.062847	0.191967	0.397212	0.482792	0.166283	0.239608	0.208616
0.279659	0.357908	0.204493	0.255884	0.312385	−0.03204	0.125815	0.304635	0.415975	0.374417	0.29152	0.339426	0.284109	0.230902
0.412124	0.286752	0.221824	0.406627	0.282239	0.311832	.	.	0.387732	0.484323	0.417963	0.336303	0.255063	0.262448
0.194126	0.130144	0.149586	0.087207	0.114223	0.230437	0.24451	0.276108	0.256223	0.24851	0.163011	0.182144	0.226637	0.168125
0.290119	0.377206	0.608886	0.525882	0.181239	0.569423	.	0.541285	0.36088	0.313619	0.338708	0.210634	0.512369	0.950232
0.14874	−0.02622	0.166472	0.154957	0.042089	0.204143	0.245693	0.13518	−0.05965	−0.00014	0.056611	0.03239	.	.
0.170904	0.145687	.	0.337794	0.227222	0.005764	0.283328	0.186359	0.166445	0.07291	0.085327	0.150756	0.403485	0.751564
0.099393	0.081865	0.091293	0.342387	.	.	.	0.239104	0.128997	0.20618	0.242395	0.218958	0.157721	0.328764
0.206839	0.176738	0.196912	0.11022	0.245976	0.143166	.	.	0.081465	0.105412	0.335423	0.142659	0.296427	.
0.083783	0.154223	0.293759	0.286406	0.197892	0.274177	0.412441	0.42552	0.137108	0.176787	0.128903	0.1434	0.255946	0.455985
0.035269	0.191671	0.009844	0.05916	.	0.109313	.	0.115447	0.140616	0.176231	0.137546	0.145131	0.148724	.
0.385279	0.205693	0.129725	0.115419	0.108826	0.669747	0.696545	0.524504	0.438118	.	0.128656	0.30831	0.139555	.
−0.11844	0.015084	0.121728	−0.01798	0.027491	−0.29823	0.267	0.022045	−0.12801	−0.16456	−0.20936	−0.01614	0.087985	0.116011

ch52
0.008369
0.000495
0.009173
.
-0.00185
-0.00042
-0.00217
-0.01457
0.00326
-0.00452
-0.00427
-0.0002
.
0.00102
0.000125
-0.01234
-0.00224
.
0.001822
.
.
0.012108
.
-0.00349

ch52
-0.06933

0.684023
0.250766
.
0.127343
0.062255
0.408377
0.081006
0.260261
0.154721
0.140605
0.319049
.
0.295961
0.076409
0.163553
0.080878
.
0.146267
.
.
-0.20812
.
0.05664

ch52
0.002034
-0.00256
0.010841
0.000084
0.00237
-0.00104
0.004986
-0.00319
0.005407
0.004485

.
-0.00097
.
-0.00191
-0.00551

ch52
0.051498
0.130019
0.199337
0.223015
0.23431
0.142834
0.58217
0.102724
0.299286
0.470771
.
0.317542
.
0.133781
0.067721
